# Emerging Role of MicroRNA-30c in Neurological Disorders

**DOI:** 10.3390/ijms24010037

**Published:** 2022-12-20

**Authors:** Manish Kumar, Guohong Li

**Affiliations:** Department of Neurosurgery, Pennsylvania State University College of Medicine, 500 University Drive, BMR, Hershey, PA 17033, USA

**Keywords:** miRNA-30c, autophagy, inflammation, ER stress, thrombosis, neurological disorders, stroke, oxidative stress

## Abstract

MicroRNAs (miRNAs or miRs) are a class of small non-coding RNAs that negatively regulate the expression of target genes by interacting with 3′ untranslated regions of target mRNAs to induce mRNA degradation and translational repression. The miR-30 family members are involved in the development of many tissues and organs and participate in the pathogenesis of human diseases. As a key member of the miR-30 family, miR-30c has been implicated in neurological disorders such as Alzheimer’s disease, Parkinson’s disease, multiple sclerosis, and stroke. Mechanistically, miR-30c may act as a multi-functional regulator of different pathogenic processes such as autophagy, apoptosis, endoplasmic reticulum stress, inflammation, oxidative stress, thrombosis, and neurovascular function, thereby contributing to different disease states. Here, we review and discuss the biogenesis, gene regulation, and the role and mechanisms of action of miR-30c in several neurological disorders and therapeutic potential in clinics.

## 1. Introduction

MicroRNAs (miRNAs or miRs) are small (18–25 nucleotides long), single-stranded, non-coding RNAs that negatively regulate gene expression mainly by binding to sequence motifs located within the 3′ untranslated regions (UTR) of their target mRNA [1]. Studies on miRs began with the discovery of the first miR (lin4) in *Caenorhabditis elegans* in 1993 and the second miR (let7) in humans in 2000 [2]. MiRs were utilized in transgenic mice in 2005 and miR inhibitors were tested in primates in 2010 [3]. Since then, a significant advance has been made in our understanding of miR biogenesis, direct target genes, mechanisms of action, and their potential for the treatment of human diseases [4,5]. One miR can regulate multiple genes, and one gene can be regulated by multiple miRs. Current research suggests that miRs represent both new clinical biomarkers and innovative therapeutic targets for many human diseases. miR-based therapeutics represent a new class of drugs for a variety of diseases, which are currently in preclinical or clinical trials, such as MRX34 (liver cancer), RG-012 (Alport syndrome), MRG-110 (wound healing, heart failure), MRG-106 (T-cell lymphoma), MRG-201 (keloid), TargomiRs (non-small cell lung cancer), and Miravirsen (hepatitis C) [6]. MiRs slightly alter the stability or expression of mRNA transcript resulting in a downregulation in levels of protein [7]. Hence, the effect of a single miR on a specific protein is small; however, because a single miR can repress the translation of several proteins, the overall effect of miR is significant [8]. A single mRNA is frequently influenced by numerous miRs and this combined impact within a regulatory network can dramatically change the outcome of a pathway.

Over 2500 miRs have been identified in the human genome, and many of them are also preserved in other species. Accumulating evidence indicates that miRs participate in various biological processes including cell proliferation, differentiation, apoptosis, and cellular metabolism, and dysregulation of miRs has been involved in the pathogenesis of various human diseases, such as cancer, cardiovascular diseases, and neurological disorders [9,10,11,12,13]. As a member of the miR-30 family, miR-30c has been implicated in metabolic, neurological, and cardiovascular diseases [14]. In this review, we summarize and discuss the biogenesis, gene regulation, and mechanistic effects of miR-30c in neurological disorders and potential therapeutic applications in clinics.

## 2. Biogenesis and Regulation of miR-30

**Figure 1:**
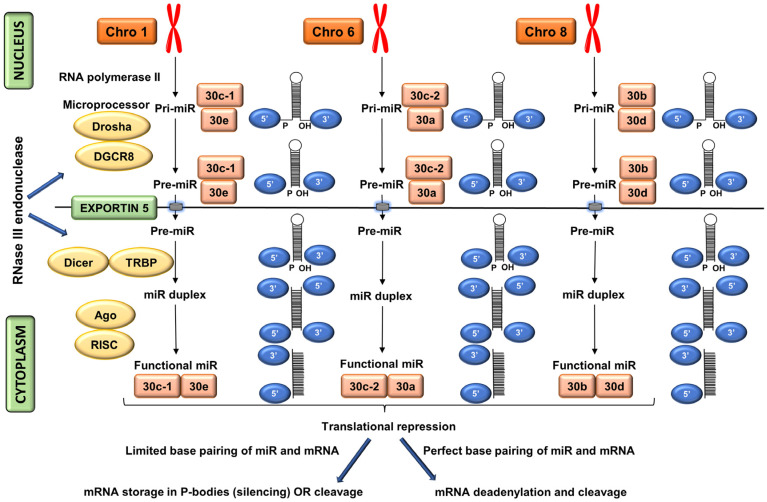
Biogenesis of miR-30 family. Ago: argonaute, Chro: chromosome, DBR1: lariat debranching enzyme, DGCR8: DiGeorge syndrome critical region gene 8, RISC: RNA-induced silencing complex, TRBP: transactivation response element RNA-binding protein.

**Figure 2:**
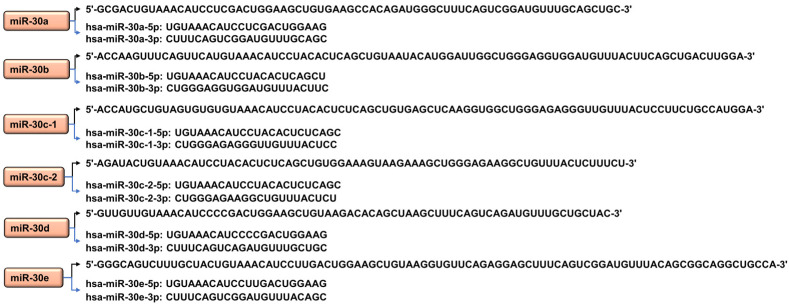
Sequences of pre-miR (black arrow) and mature miR (blue arrow) of the miR-30 family. miR-30c is also named miR-30c-5p in the literature, and miR-30c-1-5p and miR-30c-2-5p share the same mature miRNA sequence.

The miR-30 family consists of miR-30a, miR-30b, miR-30c (miR-30-c1 and miR-30-c2), miR-30d, and miR-30e members [15,16]. These are encoded within nuclear DNA in intragenic states that can be transcribed from introns either along with the host genes [17] or independently [18]. MiR-30 family members are transcribed from chromosomes 1, 6, and 8 [19] (Figure 1). These members possess great sequence resemblances including the seed sequences GUAAACA (Figure 2).

### 2.1. Nuclear Processing

**Figure 3:**
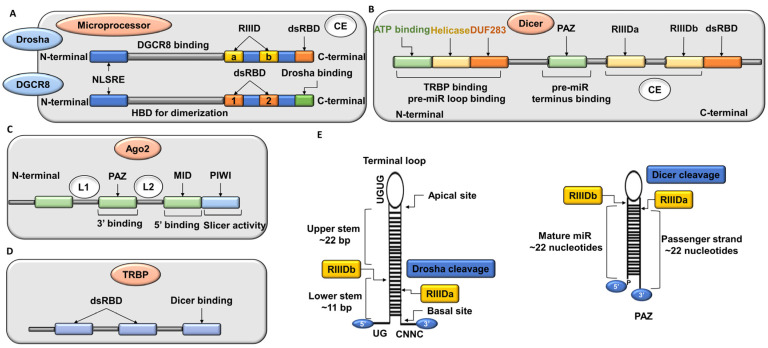
Arrangement of domains of (**A**) microprocessor (Drosha and DGCR8 complex), (**B**) Dicer, (**C**) Ago2, and (**D**) TRBP in humans. (**E**) Recognition and processing of substrate by Drosha/DGCR8 complex and Dicer (refer to Section 2.1 and Section 2.2). CE: catalytic end, HBD: heme-binding domain, L: linker.

The amino (NH) terminal of Drosha is required for nuclear localization and the carboxy (COO) terminal possesses tandem ribonuclease (RNase)-III domains (RIIIDs) and a dsRNA-binding domain (dsRBD) [20]. Cleavage by Drosha forms a minor hairpin-shaped RNA (~65 nucleotides) known as pre-miR. Along with DiGeorge syndrome critical region gene 8 (DGCR8), Drosha constitutes a “microprocessor”. This microprocessor recognizes single-stranded RNA tails, the stalk of 33–35 base pairs, and a terminal loop of the pri-miR. The microprocessor recognizes ~11 base pairs from the basal connection and ~22 base pairs from the apical connection, and Drosha splits the pri-miR at this site (Figure 3E). At least one of the different motifs on the basal and apical site of pri-miR is present in human miR. Splicing factor SRp20 (or SRSF3) attaches to the CNNC motif and speeds up the processing of human pri-miRs [21]. Additionally, the CNNC motif is necessary for the DEAD-box RNA helicase p72 (DDX17) to bind, which speeds up Drosha activity [22].

### 2.2. Cytoplasmic Processing of Pre-miR-30

The pre-miR is recognized by Dicer. The amino (NH) terminal helicase domain of Dicer enables pre-miR identification by interacting with the terminal loop. Both terminals of pre-miR are familiar to the PAZ (PIWI-Ago-ZWILLE) domain of Dicer, which has two sites for communication with the 5′-phosphorylated end and the 3′ end of the pre-miR [23]. The pre-mRNA stem is allied laterally to the protein in a system where Dicer can recognize the distance from both termini because the catalytic domains of RIIID a/b are placed ~22 nucleotides apart from the termini (Figure 3E). The guide RNA’s 5′ monophosphate is firmly linked to the 5′-phosphate-binding compartments at the junction of the MID and PIWI domains of Ago2 [5]. To reach the PAZ domain, which attaches to the 3′ end of the guide RNA, the guide RNA threads along the Ago MID-PIWI lobe’s basic channel.

### 2.3. Regulation of miR-30

**Figure 4:**
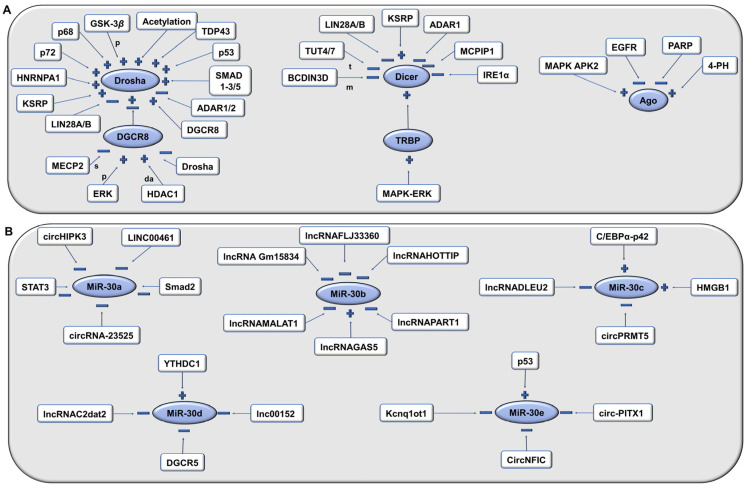
Regulation of miR-30 by (**A**) transcriptional and (**B**) post-transcriptional mechanisms. Drosha negatively regulates the expression of DGCR8 mRNA whereas DGCR8 positively upregulates the stability of Drosha, thereby maintaining a homeostatic balance. A positive sign represents an increase in pri- or pre-miR processing by positive regulation (increase in the stability and nuclear localization) of Drosha, Dicer, or Ago. A negative sign represents a decrease in pri- or pre-miR processing. ADAR 1/2: adenosine deaminases, da: deacetylation, GSK-3β: glycogen synthase kinase 3β, HDAC1: histone deacetylase 1, HNRNPA1: heterogeneous nuclear ribonucleoprotein A1, KSRP: KH-type splicing regulatory protein, m: methylation, MCPIP1: MCP-induced protein 1, MECP2: methyl-CpG-binding protein 2, s: sequestration, t: miR tailing via untemplated nucleotidyl accumulation to the 3′ end of RNA, TDP43: TAR DNA-binding protein 43, TUT 4/7: terminal uridylyl transferases.

Transcriptional and post-transcriptional mechanisms regulate the expression of miR-30. MiR-30 regulation occurs at several stages, such as during transcription, processing (e.g., Drosha and Dicer), binding to Ago proteins, and miR turnover. The cell uses a variety of tactics such as RNA-binding proteins, transcription factors, and protein and RNA enzymes (e.g., exoribonucleases and endoribonucleases) to inhibit or promote each phase [5]. A recent study indicated that YAP (yes-associated protein, Hippo pathway) regulates miR-30a via TEA (transcriptional enhanced associate) domain (TEAD) transcription factors [24]. The methylation of miR-30 genes also causes their downregulation [25]. Inhibition of EZH2 can enhance the transcription of miR-30d and miR-29b via promoter-binding activity [26]. E2F1 transcriptionally represses miR-30b expression [27]. Drosha, DGCR8, Dicer, and Ago undergo various modifications that affect miR-30 biogenesis (Figure 4A). Even the exportin5 protein can also regulate the miR-30 levels. Exportin5 is encoded by the XPO5 gene, and knockout studies of this gene revealed a decline in the levels of miRs not only in the cytoplasm but also in the nucleus [28]. This suggests that exportin5 may have other functions in addition to the transportation of pre-miR-30. Several post-transcriptional mechanisms affect the expression of miR-30 members, as shown in Figure 4B.

## 3. Role of miR-30c in Neurological Disorders

**Figure 5:**
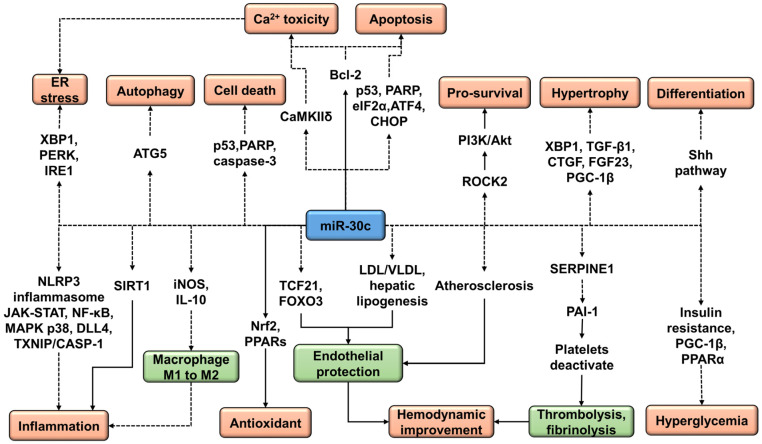
Role of miR-30c in different cell functions and signaling pathways implicated in neurological disorders. A solid arrow represents activation and a dashed arrow represents inhibition.

The existing knowledge and understanding of the neurological effects of miR-30c are still in a nascent stage. Reported data from different experimental models indicate that miR-30c targets various mechanisms and molecules that might have a major impact on the pathogenesis of stroke and other neurological disorders (Figure 5) [21,22,23,24,25,26,27,28,29,30,31,32,33,34,35,36,37,38,39,40,41,42]. In addition to cerebral ischemia reperfusion (I/R) models [43,44], a large body of data from other experimental models including human data indicates the biomarker and protective effects of miR-30c against I/R damage [45,46,47,48,49,50]. Existing evidence suggests that miR-30c attenuates cardiovascular dysfunctions [51,52,53], hyperglycemia [54,55,56], obesity [57], atherosclerosis [58,59,60], dyslipidemia [17], and hepatic lipoprotein production [61,62], which constitute major risk factors in stroke and other neurological disorders. MiR-30c potentiates pro-survival anti-apoptotic pathways [63,64,65]. Experimental findings indicated that in the initial stages of ischemia suppression of autophagy by miR-30c may worsen neurodegeneration in the spinal cord I/R injury model [66]. Although autophagy increases initially in I/R, it later ceases due to lysosomal dysfunction, and both are considered to be protective responses against ischemic insult. Nevertheless, many studies corroborate that uninhibited late-phase chronic autophagy is primarily responsible for the post-stroke increase in the oxidative stress and infarct area via self-digestion, which can be attenuated by inhibition of autophagy [67,68], and it is possible to minimize I/R injury by controlling autophagy via miR-30c. Furthermore, matrix metalloproteinases are chiefly responsible for the deterioration of the blood–brain barrier in stroke, and existing data suggest that miR-30c can suppress the matrix metalloproteinase (MMP)-9 activity [69]. There is a high possibility that miR-30c modulation might alter the course of pathogenesis in neurological disorders; however, cell- and disease-specific and rationalized experimental regimens are necessary to further establish the role of miR-30c in central nervous system disorders.

### 3.1. MiR-30c in Stroke

At present, the lack of sufficient clinical data severely hampers our knowledge about the exact role and tissue- or cell-specific expression profile of miR-30c in stroke. Tan et al. [70] showed an increase in the expression of all the miR-30 family members in the blood samples and small arteries of young stroke patients. A report by Sepramaniam et al. [71] indicated the downregulation of miR-30c in the blood sample of young as well as upper-age stroke patients. This decrease in expression of miR-30c is associated with the acute phase of stroke rather than the recovery phase after the stroke which also suggests the biomarker potential of miR-30c in stroke. However, a large body of pre-clinical data indicates that downregulation of miR-30c-5p expression in the brain potentiates degenerative mechanisms, resulting in an increase in infarct area [43,44,72]. In a study, “miR-30c mimic” pretreatment caused hippocampal neuroprotection, neurological improvement, and a decrease in infarct area in rats against transient middle cerebral artery occlusion. Data suggested that “miR-30c mimic” enhanced cell survival and attenuated apoptosis via neurotrophic factors such as brain-derived neurotrophic factor and neurotrophin-3. This upregulation of neurotrophic factors is attributed to the direct targeting of the SRY-box transcription factor 9 (SOX9) gene by “miR-30c mimic” in HT22 cells under oxygen–glucose deprivation and reperfusion [43]. The previous observations substantiate these findings showing that SOX9 deletion in mice imparted a protective effect against transient middle cerebral artery occlusion injury, resulting in improved post-stroke neurological recovery and neuroprotection [73]. From in vitro oxygen–glucose deprivation and reperfusion models, it is evident that miR-30c targets 3′ UTR of SOX9 [44], homeodomain-interacting protein kinase 1 (HIPK1) [72], and Rho-associated coiled-coil containing protein kinase 2 (ROCK2) [74] gene, which might attenuate oxidative stress and inflammation. Experimental data revealed that “miR-30c-5p antagomir” enhanced oxidative stress, inflammation, infarct area, and neurological deficits in a transient middle cerebral artery occlusion rat model. These effects were prevented by the upregulation of miR-30c-5p by sevoflurane or flurbiprofen axetil pretreatments [44,72]. MiR-30c-5p attenuates pathological pathways such as mitogen-activated protein kinases by targeting ROCK2 [74], which presents a novel therapeutic target, miR-30c/ROCK2, in the pathogenesis of stroke.

More than 100 circulating miRs have been found in sera from healthy human subjects [75]. Circulating miRs exhibit extraordinary stability and resistance to destruction by endogenous RNase activity [76]. All the members of the miR-30 subfamily including miR-30c are highly expressed in platelets [77] and are involved in blood coagulation and platelet activation [54]. Multiple brain conditions, including ischemic stroke and thrombotic vascular events, have been linked to the overexpression of plasminogen activator inhibitor-1 (PAI-1) in the plasma, endothelial cells, and platelets [78]. MiR-30c directly targets the PAI-1 via the SERPINE1 gene [79,80,81] including the osteoprotegerin (OPG) gene [82], which mitigates atherosclerosis, thrombosis, and embolism, which are major causes of clogging of arteries [55]. MiR-30c-5p can protect against endothelial damage by targeting TGF-β1 [83] and TCF21 [58] and attenuating endothelial pyroptosis by downregulating Forkhead Box O3- NOD-, LRR-, and pyrin domain-containing protein 3 (FOXO3-NLRP3) inflammasomes [59] and α2-antiplasmin [84]. Reduced circulating miR-30c-5p levels are linked to intima-media thickness, early atherosclerosis, and plaque formation [59,60], which are important risk factors in ischemic stroke. In an in vitro prototype of sprouting angiogenesis, Bridge et al. [85] demonstrated that the excessive appearance of miR-30 members in endothelial cells augmented vessel number and length. MiR-30b and miR-30c target delta-like canonical Notch ligand 4 (DLL4), a Notch signaling family membrane-bound ligand, which is essential for vascular development and angiogenesis, encouraging the repair of damaged vessels and maintaining uninterrupted cerebral blood flow. These findings suggest that miR-30c targets many biomolecules and genes that can improve cerebral blood flow by protecting endothelial cells and attenuating arterial occlusion.

### 3.2. MiR-30c in Parkinson’s Disease

A majority of clinical findings highlight that miR-30c-5p is significantly downregulated in the serum and peripheral blood mononuclear cells of Parkinson’s disease (PD) patients [86,87,88]. Marques et al. [89] reported no significant change in miR-30c-5p expression in the cerebrospinal fluid of PD patients. In a study, treatment with levodopa increased the expression of miR-30a, miR-30b, and miR-30c in the blood of PD patients in association with improvement in motor functions such as rigidity, tremor, and bradykinesia [90]. These findings highlight the biomarker potential of miR-30c in PD. However, a few reports indicate an increase in the expression of miR-30c in substantia nigra, cerebrospinal fluid, plasma extracellular vesicles, and blood of PD patients [91,92]. Therefore, still more data are required to establish a clear link between the expression of miR-30c and PD in human subjects.

In PD, the aggregation of misfolded proteins, injured mitochondria, and reactive oxygen species leads to endoplasmic reticulum (ER) stress and unfolded protein response (UPR). MiR-30c-2-3p is considered a biomarker of UPR. In a cellular PD model, expression of miR-30c-2-3p was significantly enhanced in dopaminergic SHSY5Y cells under ER stress. Treatment with DADLE (D-Alanine 2, D-Leucine 5 Enkephaline), a synthetic delta opioid peptide, enhanced cell survival in dopaminergic SHSY5Y cells against ER stress by suppressing UPR by reducing the expression of miR-30c-2-3p [93]. The findings indicated that the downregulation of miR-30c-2-3p might attenuate the UPR-ER stress mechanism in association with the activation of delta opioid receptors (DOR). Autophagy dysfunction is consistently linked with an increase in levels of α-synuclein, damaged or dysfunctional proteins, and motor dysfunctions [94]. Parallel to these findings, suppression of autophagy by miR-30c-5p enhanced dopaminergic loss and decreased dopamine levels in substantia nigra pars compacta in a mouse PD model [30]. In vivo and in vitro studies showed that miR-30c-5p antagomiR attenuated dopaminergic apoptosis and oxidative stress and improved the behavioral symptoms (e.g., rotarod performance, forelimb grip strength) of PD by targeting autophagy via the ATG5 gene. These findings suggest that reactivation of autophagy by targeting miR-30c-5p might be a viable strategy for treating PD-related neurodegeneration.

In silico tools predict several genes (e.g., PARK1-18, LRRK2, GIGYF2, UCHL1, SQSTM1, SLC1A4, SNCA, VPS35, and PITX3) and pathways targeted by miR-30c in PD [86,90]. Martins et al. [88] indicated that miR-30c might regulate α-synuclein by modulating the protein ubiquitination pathway by targeting USP37, UBE2J1, NEDD4, and UBE2I genes. Although previous studies showed that miR-30c has a major impact on dopaminergic signaling via ER stress and autophagy, miR-30c regulates many pathways of oxidative stress and inflammation that could alter the course of PD progression.

### 3.3. MiR-30c in Multiple System Atrophy

Multiple system atrophy (MSA) is a progressive neurological condition characterized clinically by parkinsonism, cerebellar ataxia, autonomic dysfunction, and various motor and non-motor symptoms. The pathogenesis of MSA shows neurodegeneration in striatonigral and/or olivopontocerebellar systems accompanied by intracellular aggregates of α-synuclein. At the initial stage of the disease, there is considerable overlap of symptoms between MSA and PD, but without a distinct differentiating biomarker between these two disorders. However, the expression of serum miR-30c-5p was found to be significantly upregulated in MSA patients relative to both PD patients and healthy controls [95]. The findings indicated that miR-30c-5p might be a possible differentiating factor between MSA and PD with biomarker potential.

### 3.4. MiR-30c in Alzheimer’s Disease

Alzheimer’s disease (AD) manifests neurodegenerative pathogenesis with an expression of extracellular amyloid-β and intraneuronal hyperphosphorylated tau as the prominent hallmarks. Several miRs including miR-30c are implicated in AD. Analysis of miRs derived from small RNA sequencing in the blood sample of aged AD patients (age 70.3 ± 7.9 years) by using “omiRas” revealed an increase in miR-30c-5p [96]. This substantiates their biomarker potential in AD. In the cholesterol-fed LOAD (late-onset AD) rabbit model, findings indicated an increase in miR-30c in the cortex of the brain [97]. The upregulation of miR-30c in AD has been consistent with human data. DIANA miRPath identified that miR-30c-5p targets many pathways, such as synaptic plasticity, long-term potentiation, ubiquitin-mediated proteolysis, axon guidance, Wnt/ErbB signaling pathway, and aldosterone-regulated sodium reabsorption in AD [96]. A few reports suggested upregulation of miR-30a-5p [98] and miR-30b [99] in blood and brain in AD, which affected synaptic plasticity, neuron survival, growth factors, and signal transduction pathways (e.g., Notch1) [100,101,102]. Therefore, in addition to miR-30c, literature findings suggest the participation of different miR-30s in the pathogenesis of AD; however, their exact role is still unclear. Data from different experimental models suggest the beneficial potential of miR-30c in AD [103]. MiR-30c promotes nerve repair against peripheral nerve injury [104] and neurogenesis in the olfactory bulb and hippocampus [105] which are primarily affected in AD. Hyperglycemia and dyslipidemia are the major risk factors in LOAD that also potentiate low-grade inflammation marked by an increase in pro-inflammatory cytokines and oxidative stress. In vitro studies using human umbilical vascular endothelial cells (HUVECs) showed that miR-30c-5p can reduce the inflammatory response, such as nuclear factor kappa light-chain-enhancer of activated B cells (NF-κB) and oxidative stress against oxidized low-density lipoprotein [106] by targeting the TCF7 gene [107], phosphatase and tensin homolog (PTEN) [108], and the Wnt7b/β-catenin pathway [109]. MiR-30c can attenuate high glucose-induced oxidative stress and inflammation by targeting the Lmp7 gene in SV40-MES13 cells [110]. From these findings, it is evident that miR-30c has the potential to arrest the pathogenesis of AD by targeting various pro-survival mechanisms. However, the specific role of miR-30c in the buildup of neurotoxic aggregates, synaptic modulation, long-term potentiation, and kinase activity is still to be explored by using AD-specific models.

### 3.5. MiR-30c in Epilepsy

Epilepsy is a chronic neurologic condition defined by spontaneous recurring seizures caused by abnormal neuronal excitability. MiRs regulate the biogenesis of many proteins during and after epileptic episodes [111]. This implies that miRs may influence neuronal excitability and remodeling responses. Nudelman et al. [112] first showed a close relationship between a change in miR expression and seizures. In a study, miR-30c expression was upregulated in the acute and chronic phases and downregulated in the latent phase of pilocarpine-induced temporal lobe epilepsy [113]. It is noticeable that epilepsy can result in miR dysregulation. Preclinical research suggests that pathways implicated in miR dysregulation, including the apoptotic, inflammatory, and growth factor signaling mechanisms, may be potential new approaches for the diagnosis and treatment of epilepsy. Zhou et al. [114] showed significant downregulation of miR-30c in the hippocampus of valproic acid (anti-epileptic drug)-treated rats. The modulation of miR-30c by valproic acid suggested possible involvement in neurite outgrowth, neurogenesis, inhibitory neurotransmission (γ-amino butyric acid), and different pathways (e.g., ERK, phosphatidylinositol 3-kinase/Akt, and Wnt/β-catenin). However, substantial data are required to further strengthen this relationship. Hence, more insights into the molecular mechanisms associated with epileptic disease progression and miR-30c are essentially required.

### 3.6. MiR-30c in Huntington’s Disease

Huntington’s disease (HD) is a neurological disorder manifested by an extension of cytosine, adenine, and guanine (CAG) repeats (polyglutamine) in the gene encoding the Huntington protein. Classically, the symptoms show chorea and loss of coordination as prominent motor defects, including psychiatric and cognitive anomalies. Existing data from HD patients indicate a significant upregulation of miR-30c (along with miR-30a, miR-30b, and miR-30e) in the frontal cortex and striatum (dorsal caudate) brain regions [115]. However, more studies are needed to establish the mechanistic link of miR-30c in the pathogenesis of HD.

## 4. Bioinformatics Prediction of miR-30c Targets

**Figure 6:**
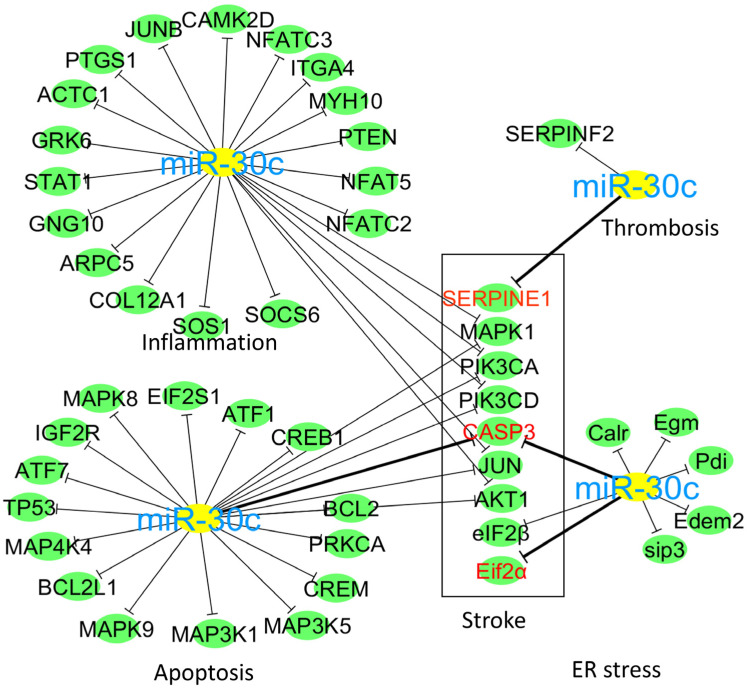
Prediction of miR-30c target genes. The green nodes are the predictive interactive genes of miR-30c involved in thrombosis, inflammation, ER stress, and apoptosis pathways, and the red nodes are the selected target genes common with neurological disease.

Altered miR expression is associated with neurological and other diseases. It is now possible to modulate miR functions by administration of synthetic miR mimics or inhibitors, similar to how antisense RNAs and RNA inhibitors are employed (extensively used methods for examining the role of a gene and in gene therapy). Prediction algorithms can predict potential miR targets based on complementary RNA sequences in the 3′UTR of miR target genes (Figure 6). We identified several miR-30c target genes potentially associated with neurological disorders using a bioinformatic tool with an online target prediction algorithm, miRWalk (http://mirwalk.umm.uni-heidelberg.de/, accessed date: 10 January 2021). Gene targets for miR-30c-5p were selected through Gene Ontology (GO) functional analysis and through Ontology Biological Process (GOBP). Biological networks were created using Cytoscape v3.2.1 software (https://cytoscape.org/release_notes_3_2_1.html).

## 5. Future Directions and Conclusions

In this review, we summarize and discuss the current progress of miR-30c in the pathogenesis of several neurological disorders and its therapeutic potential. Bioinformatic analysis and experimental evidence suggest that miR-30c regulates many genes as targets that are associated with a number of pathogenic processes, such as autophagy, apoptosis, ER stress, inflammation, oxidative stress, thrombosis, and neurovascular function, thereby contributing to the pathophysiology of different neurological diseases. As miR is abundantly expressed in the brain, the cell type-specific expression and spatiotemporal regulation of miR-30c with specific neurological disorders remain poorly understood. Moreover, given that miR-30c regulates many related mRNAs, the multiplicity of its binding targets poses potential difficulties in obtaining specificity of targeting, leading to unexpected side effects. More research remains to be conducted in developing effective therapeutic targeting strategies to enable site-specific drug delivery, reducing off-target effects, and decreasing unwanted effects, thereby enhancing a miR drug’s therapeutic efficacy. MiR-30c plays a beneficial role in neuropathology via regulating autophagy, neuroinflammation, thrombosis, ER stress, oxidative stress, amyloid-β deposition, and hyperphosphorylation of tau protein, providing a reason for further exploration. Therapeutic targeting of miR-30c by using synthetic mimics or inhibitors for stroke and other neurological diseases is under investigation in our lab.

## Data Availability

Not applicable.
